# Supplementary material for: Treatment of Hodgkin Lymphoma Relapsed after Autologous Stem Cell Transplantation
Source: J Clin Med. 2020 May 8;9(5):1384. doi: 10.3390/jcm9051384 (PMC7290321; doi:10.3390/jcm9051384)
Supplement: Supplementary file 1 [file jcm-09-01384-s001.pdf]

Supplementary file

Table S1. Novel agents in relapsed/refractary(R/R) Hodgkin Lymphomas (HL) after autologous stem cell transplantation (Auto-HCT).

Agent	Phase	N	ORR, %	CR, %	Median duration of response	PFS
**Single agent**						
Brentuximab Vedotin [7]	2	102	75%	34%	20.5 for those patients in CR	Median PFS 5.6 months
AFM-13[18]	1	28	11.5-23%	0%	-	-
Nivolumab[8]	2	243	69%	15%	16.6 months	Median PFS 14.7 months
Pembrolizumab[9]	2	210	71.9%	27.6%	16.5 months	6 month PFS 72%
Bendamustine [30]		67	57%	25%	-	Median PFS 10 months
Panobinostat[35]	2	129	23%	4%	6.9 months	Median PFS 6.1 months
Ruxolitinib [39]	2	33	9.4%	0%	7.7 months	Median PFS 3.5 months
ADCT-301[20]	1	67	81%	50%	7.7 months	Median PFS 6.7 months
CD30 CAR T cells[41]	1/2	22	64%	53%	-	Median PFS 164 days
**Combinations**						
Brentuximab plus Bendamustine [33]	1/2	65	78% (phase 2 dose)	-	-	-
BV and ipilimumab[27]	1	21	67%	55%	-	1-year PFS 60%
BV and nivolumab[28]	1	18	95%	65%	-	1-year PFS 68%
BV plus ipilimumab and nivolumab [29]	1	22	95%	84%	-	1-year PFS 72%
AFM13-Pembrolizumab[19]	1b	30	87%	35%		
Panobinostat-ICE [36]	2	11	-	82%	-	-

Abbreviations: HL: Hodgkin's lymphoma; BV: Brentuximab vedotin; Auto-HCT: Autologous hematopoietic stem cell transplantation; N: number; ORR: Overall response rate; CR: Complete response; PFS: Progression-free survival.

Treatment algorithim for Relapsed or Refractory Hodgkin Lymphoma after Auto-HCT

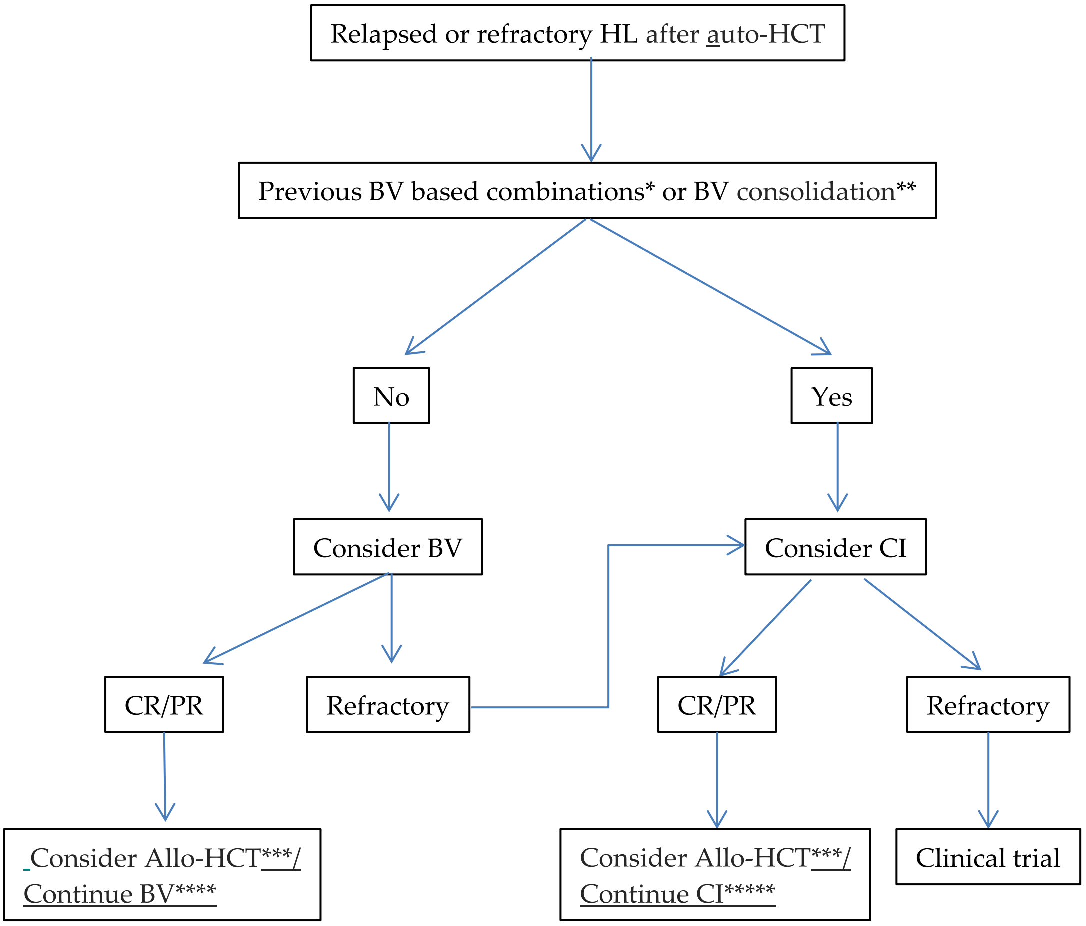

Figure 1. Treatment algorithim for Relapsed or Refractory Hodgkin Lymphoma after Auto-HCT.

Abbreviations: HL: Hodgkin's lymphoma; BV: Brentuximab vedotin; Auto-HCT: Autologous hematopoietic stem cell transplantation; CI: Checkpoint inhibidors; CR: Complete response; PR: Partial response; Allo-HCT: Allogeneic hematopoietic stem cell transplantation.

* Includes first line treatment with BV-AVD, salvage therapies including platinum based regiments (ICE, DHAP, or ESHAP), gemcitabine-based regimens and checkpoint inhibidors in combination with BV.

** Consolidation with BV following the AETHERA trial.

*** Always taking into consideration risk factors of the patient, disease and transplantation procedure.

***** Continue BV until 16 cycles or toxicity.

***** Continue CI until progression or toxicity. Consider stopping CI if a CR is achieved.
